# Comprehensive molecular characterization of treatment-free remission and molecular relapse in chronic myeloid leukemia patients: the EURO-SKI Biomarker Study

**DOI:** 10.1038/s41375-026-03001-5

**Published:** 2026-06-18

**Authors:** S. Rinaldetti, M. Chepeleva, A. Hedblom, C. Sticht, M. Muciek, P. Panayiotidis, J. Richter, U. Olsson-Strömberg, D. Nowak, V. Nowak, P. Ljungman, N. von Bubnoff, K. Lotfi, L. Stenke, T. H. Brümmendorf, H. Hjorth-Hansen, T. Gedde-Dahl, W. Majeed, A. Burchert, P. V. Nazarov, E. Felde, I. Tarnopolscaia, C. Hoelting, A. Fabarius, W.-K. Hofmann, F.-X. Mahon, S. Saussele, M. Pfirrmann

**Affiliations:** 1 Department of Hematology and Oncology, Centre Hospitalier de Luxembourg, Luxembourg, Luxembourg; 2 Department of Hematology and Oncology, Medical Faculty Mannheim, Heidelberg University, Mannheim, Germany; 3 Multiomics Data Science, Department of Cancer Research, Luxembourg Institute of Health, Luxembourg, Luxembourg; 4 Faculty of Science, Technology and Medicine, University of Luxembourg, Esch-sur-Alzette, Luxembourg; 5 Division of Experimental Cancer Research, Department of Translational Medicine, Clinical Research Centre, Lund University, Malmö, Sweden; 6 Department of Bioinformatics, Medical Faculty Mannheim, Heidelberg University, Mannheim, Germany; 7 Medical Research Center, Medical Faculty Mannheim, University of Heidelberg, Mannheim, Germany; 8 Laikon General Hospital, National and Kapodistrian University of Athens, Athens, Greece; 9 Department of Molecular Medicine and Gene Therapy, Lund University, Lund, Sweden; 10 Department of Medical Science and Division of Hematology, Uppsala University Hospital, Uppsala, Sweden; 11 Department of Cellular Therapy and Allogeneic Stem Cell Transplantation, Karolinska University Hospital, Karolinska Comprehensive Cancer Center, Solna, Sweden; 12 Department of Medicine Huddinge, Karolinska Institutet, Stockholm, Sweden; 13 Department of Hematology and Oncology, University Medical Center Schleswig-Holstein, Schleswig-Holstein, Germany; 14 University Cancer Center Schleswig-Holstein, Campus Lübeck, Lübeck, Germany; 15 Department of Hematology, Linköping University Hospital, Linköping, Sweden; 16 Department of Hematology, Karolinska University Hospital and Karolinska Institutet, Stockholm, Sweden; 17 Department of Hematology, Oncology, Hemostaseology and Stem Cell Transplantation, Faculty of Medicine, RWTH Aachen University Hospital, Aachen, Germany; 18 Center for Integrated Oncology, Aachen Bonn Cologne Düsseldorf (CIO ABCD), Aachen, Germany; 19 St Olavs Hospital HF, Trondheim, Norway; 20 Department of hematology and Institute for clinical medicine, Oslo University Hospital and University of Oslo, Oslo, Norway; 21 Department of Hemato-Oncology, Stavanger University Hospital, Stavanger, Norway; 22 Department of Internal Medicine, Hematology, Oncology and Immunology, University Hospital Marburg, Marburg, Germany; 23 Bioinformatics and AI, Department of Medical Informatics, Luxembourg Institute of Health, Luxembourg, Luxembourg; 24 Département d’Hématologie, Institut Bergonié, University of Bordeaux, Inserm, Bordeaux, France; 25 Institut für Medizinische Informationsverarbeitung, Biometrie und Epidemiologie (IBE), Medizinische Fakultät, LMU München, München, Germany

**Keywords:** Translational research, Chronic myeloid leukaemia

## Abstract

The European Stop Kinase Inhibitors (EURO-SKI) trial was launched to investigate the discontinuation of tyrosine kinase inhibitors (TKIs) in patients with chronic myeloid leukemia (CML). The molecular mechanisms underlying sustained treatment-free remission (TFR) and loss of TFR, are still poorly understood. To address this, we performed whole transcriptome gene expression analyses coupled with NanoString nCounter-based absolute gene quantification. Gene expression was assessed in peripheral blood leukocytes from 240 CML patients on the final day of TKI intake (training sample: *n* = 122, validation sample: *n* = 118) as well as from 10 healthy controls. To identify TKI-specific mechanisms, transcriptomic data from an external dataset of 96 TKI-naïve CML patients was incorporated into our analyses. TFR patients showed an activation of GATA1, KLF1 and MYBL1 regulons characteristic for erythroid progenitor cells. TFR patients maintain persistent communication between innate (natural killer and dendritic cells) and adaptive immunity (e.g., CD8+, CD4+, and γδ T lymphocytes), mirroring patterns observed in healthy controls. This intercellular communication is disrupted in patients with loss of TFR. Furthermore, we identified an FLT3 threshold that distinguished two patient groups with significantly different probabilities of TFR loss. Leveraging mechanisms that reestablish or reinforce the communication between innate and adaptive immunity could be pivotal in achieving durable TFR.

## Introduction

The European Stop Kinase Inhibitor (EURO-SKI) study is the largest trial to evaluate tyrosine kinase inhibitor (TKI) discontinuation in chronic myeloid leukemia (CML) [1, 2]. Nearly half of patients remain in treatment-free remission (TFR) gafter stopping TKIs. However, molecular relapse defined as loss of TFR (i.e. loss of major molecular remission [MMR]) after TKI discontinuation, remains still a significant concern. The identification and validation of reliable prognostic biomarkers for successful TFR continues to be an important area of ongoing research. In recent years, the elucidation of immune mechanisms governing TFR and relapse has emerged as one of the most daunting challenges. A plethora of mechanisms have since been described [3, 4]. It became clear that the TFR/relapse ecosystem is driven by a complex interplay among multiple immune effector cells [5–8]. The EURO-SKI Biomarker study offers an in-depth molecular characterization of patients in TFR and relapse, supporting the identification of key mechanisms underlying TFR/relapse detectable in peripheral blood. Our goal was to decipher the key regulators and pathways associated with TFR/relapse and to explore novel biomarkers via comprehensive gene expression analyses on both a training and an independent validation sample.

## Materials/subjects and methods

### Study design and participants

The prospective multicenter tyrosine kinase inhibitor (TKI) discontinuation trial (EURO-SKI, NCT01596114) enrolled adult chronic-phase patients with stable deep molecular response (DMR: BCR::ABL1 transcripts ≤0.01% on the International Scale, IS) for at least 1 year while on TKI treatment for at least 3 years. In the EURO-SKI trial, TFR was defined as the sustained maintenance of MMR (BCR::ABL1 transcripts ≤0.1% on the IS) over time.

**Fig. 1:**
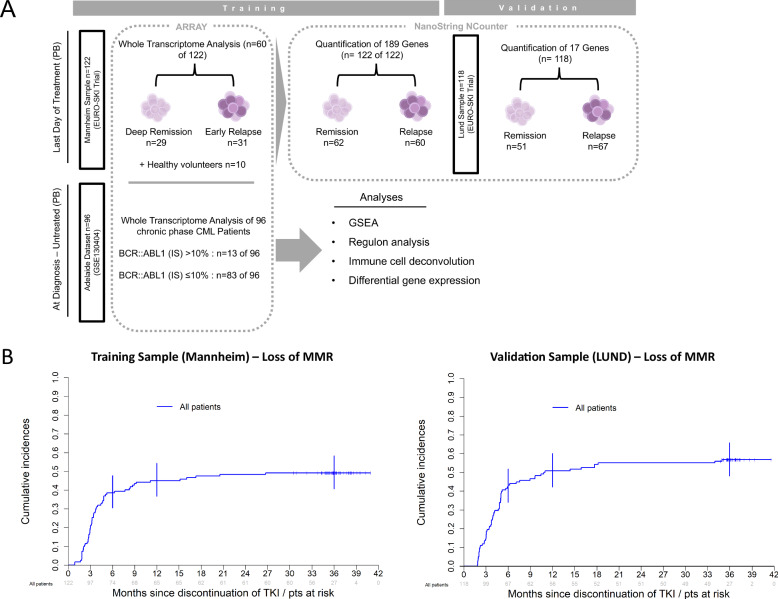
EURO-SKI Biomarker Study design and outcome. **A** The EURO-SKI Biomarker study design included a training sample (Mannheim) and a validation sample (Lund). The Adelaide dataset was integrated into several downstream analyses to compare transcriptomic changes between patients at diagnosis versus patients at the last day of tyrosine kinase inhibitor (TKI) intake. Designed with Biorender. **B** Cumulative incidences of MMR loss (=relapse) after TKI discontinuation were analyzed in 122 patients of the training sample and 118 patients of the validation sample. The curve was estimated using the cumulative incidence function, with death and TKI restart prior to MMR loss considered as competing events. Vertical bars at 6, 12, and 36 months indicate the upper and lower limits of the 95% confidence intervals for the estimated incidence. MMR: Major molecular response, Pts Patients, PB Peripheral blood.

The EURO-SKI Biomarker Study included 240 of the 728 EURO-SKI patients and 10 healthy volunteers. In accordance with the Declaration of Helsinki, written informed consent was obtained from all patients. Peripheral blood samples were collected from CML patients on the last day of TKI intake (=baseline) and shipped within 24 h to designated standardized EUTOS laboratories. RNA was isolated from peripheral blood leucocytes for molecular response assessment via RT-PCR and for further transcriptomic gene expression analyses. We included transcriptomic data from two in silico datasets with array expression data at the time of CML diagnosis: the Adelaide dataset (GSE130404) and the Seattle dataset (GSE4170) (Fig. 1A) [9, 10].

### Gene expression analyses

Clariom D arrays (Applied Biosystems, Massachusetts) were used for whole transcriptome analyses. The Transcriptome Analysis Console (TAC) software (version 4.0.3.14) was used for preprocessing, employing the Signal Space Transformation-Robust Multi-Chip Analysis (SST-RMA) method. This approach incorporates background correction, GC correction, quantile normalization, and probe-set summarization. Differentially expressed genes between relapse patients and those in TFR were identified using the *limma* R package [11]. The method fits a linear model for each gene and computes moderated t-statistics using an empirical Bayes method. *P*-values were adjusted for multiple testing using the Benjamini–Hochberg method, and a threshold of *P* < 0.05 was used to determine statistical significance. Gene signatures were tested with *GSEA* (Gene Set Enrichment Analysis Version 4.3.3, Broad Institute) [12].

The NanoString nCounter assay (Bruker, Massachusetts) was normalized using the geometric means of reference genes (*ABL1, GAPDH, GUSB, RPL19, TUBB)* and 6 internal positive controls. Negative background subtraction was performed by 5 negative internal controls. We used *nSolver 4.0* for data preprocessing and quality control. Batch correction was performed using *ComBat* [13]. Batch correction was applied to both the Clariom D and NanoString datasets to address technical variance between samples within each dataset. This was used only to mitigate intra-dataset noise and not to merge independent cohorts (Supplementary Fig. S1A).

### Regulon analysis

To infer protein activity from bulk gene expression profiles, we performed master regulator analyses. To ensure a robust and comprehensive reconstruction of regulatory logic, we built context-specific transcription factor (TF)-target gene networks (regulons) from multiple sources using the *ARACNe-AP* algorithm (*ARACNe* = Algorithm for the reconstruction of accurate cellular networks) [14]. Two networks were reconstructed using independent, publicly available CML studies (Adelaide dataset: GSE130404, Seattle dataset: GSE4170) to capture disease-specific interactions. These were complemented by a pre-compiled regulon network from TCGA acute myeloid leukemia (AML *ARACNe*) patients to incorporate a broader view of myeloid biology.

Next, the activity of each TF was calculated for the Mannheim sample using the *msVIPER* algorithm [15]. This algorithm assesses the enrichment of a regulon’s target genes within a gene expression signature. The signature was generated by comparing gene expression between the relapse and TFR patient groups using a moderated *t*-test. Statistical significance was assessed against a null model generated from 1000 sample permutations. The analysis produced a list of regulons ranked by their differential activity, reported as a Normalized Enrichment Score (NES), between the relapse and TFR groups.

### Transcriptome-based immune profiling

The relative abundance of 36 immune and stromal cell types was inferred from whole transcriptome gene expression profiles using the *xCell* tool [16]. The analysis was performed via the *immunedeconv* R package, with settings optimized for microarray data [17]. Scores generated by *xCell* were correlated with MCP-counter estimates to confirm the consistency of relevant immune lineages (Supplementary Fig. S1B and S1C) [18]. To identify differentially abundant cell populations, pairwise comparisons across the groups were performed using the Wilcoxon rank-sum test. The resulting p-values were adjusted for multiple comparisons within each cell type using the Benjamini–Hochberg method.

To investigate the interplay between immune cell populations, we performed a correlation analysis on the *xCell* enrichment scores. For each patient group (TFR, Relapse, Healthy), a pairwise correlation matrix was computed between all cell types using Spearman’s rank correlation coefficient.

### Independent component analysis

To identify and characterize data-driven transcriptional programs in an unsupervised manner, we performed consensus Independent Component Analysis (ICA) using the *consICA* R package [19], extracting 20 components. This analysis decomposed the gene expression matrix into two outputs: a matrix of independent components (IC) with gene contributions and a matrix of component activities (weights) across blood samples. For functional annotation of ICs, genes with a statistically significant contribution to a component (FDR < 0.05) were subjected to Gene Ontology (GO) enrichment analysis. To contextualize these transcriptional programs, the activity of each component was correlated with xCell-derived immune scores using Spearman’s rank correlation.

### Statistical analysis

TFR was measured from the date of TKI discontinuation to the observation of the first event, i.e. loss of MMR, death, or restart of TKI without prior loss of MMR, whichever occurred first. While loss of MMR was the primary event of interest, the other two events were treated as competing events for the observation of MMR loss. In the absence of any events, observation time was censored at the date of the last molecular follow-up.

Considering the competing events, cumulative incidence probabilities of MMR loss were obtained using the Aalen-Johansen estimator and compared by the Gray test [20, 21].

In the training sample, the influence of potential prognostic genes on the hazard of MMR loss was analyzed using a cause-specific Cox model, with the two competing events censored at the time of their occurrence. In addition to genes, candidate prognostic factors in multiple modeling were age, sex, duration of TKI therapy or duration of DMR prior to treatment discontinuation. For variable selection, the Akaike information criterion was applied. To identify a reliable cut-off for the genes included in the multiple model, we performed bootstrap resampling. From the training dataset, we generated 1000 bootstrap samples of the same size (*n* = 122). For each sample, we identified the threshold associated with the smallest *p*-value, adjusting *p*-values for multiple testing. This minimal *p*-value approach was used to determine cutoffs that defined two groups with the most distinct cumulative incidences of MMR loss, as evaluated by Gray’s test [22].

All significant prognostic findings identified in the training sample were subsequently evaluated in the validation sample. In the confirmatory testing of genes in the validation sample, multiple testing was adjusted by the Benjamini–Hochberg procedure.

Differential gene expression was compared between groups using the Mann–Whitney U test. Besides the use of the minimal *P*-value approach and the testing of the prognostic findings in the validation sample, for the two-sided *P*-values the unadjusted significance level of 0.05 was applied at all statistical tests.

## Results

### Patient characteristics

Gene expression analyses were conducted on 240 CML patients at the final day of TKI administration. Blood samples were obtained from the University Medical Centre Mannheim (*n* = 122), designated as the training sample, and from Lund University (*n* = 118), defined as the validation sample (see Fig. 1A). Initially, exploratory whole transcriptome gene expression analyses were carried out on 60 CML patients from the training sample. To examine differences in gene expression between TFR and remission patients, 29 individuals with long-term DMR (median DMR duration: 9.4 years) and deep molecular remission (BCR::ABL1 < 0,01% or BCR::ABL1 negative) were selected, along with 31 patients who experienced loss of MMR within the first three months following TKI discontinuation. In a next step, a curated panel of 189 genes was quantified via the NanoString nCounter in all 122 patients of the training sample (= Mannheim sample, Fig. 1A). Most participants received imatinib treatment only (23% received a second-generation TKI in first- or second-line), with a median treatment duration of 7.6 years and a median DMR duration of 4.3 years (Table 1). Subsequently, a reduced gene panel was evaluated in the validation sample (= Lund sample), which exhibited clinical characteristics comparable to those of the training sample. The median duration of TKI treatment was 7.7 years, and the median duration of DMR was 4.5 years. In both the training and validation sample, the majority of relapses after TKI discontinuation occurred within the first 6 months. The cumulative incidence of loss of TFR after 36 months of TKI discontinuation was 49% in the training sample (95% confidence interval: 41–58%) and 57% in the validation sample (95% confidence interval: 48–66%, Fig. 1B, C).

**Table 1:** Characteristics of the EURO-SKI patients in the training and validation sample at the time of tyrosine kinase inhibitor (TKI) discontinuation.

Characteristics	Training sample (*n* = 122)	Validation sample (*n* = 118)
Gender, males, number (percent)	66 (54)	70 (59)
Median age at diagnosis, years (range)	51 (19–77)	53 (17–80)
Median percentage of blasts in peripheral blood at diagnosis (range)^a^	1 (0–18)	1 (0–15)
Median number of platelets at diagnosis, 10^9^/L (range)^b^	510 (72–1391)	471 (107–2674)
Median number of spleen size below costal margin at diagnosis, cm (range)^c^	0 (0–32)	0 (0–20)
Median age at TKI stop, years (range)	61 (23–84)	62 (22–84)
Median time from diagnosis to TKI stop, years (range)	7.6 (3.2–21.5)	7.7 (3.1–16.2)
Median duration of TKI therapy, years (range)	7.5 (3.0–14.0)	7.4 (3.0–12.6)
Median duration of DMR before stop, years (range)	4.3 (1.0–12.8)	4.5 (1.2–11.7)
Transcript type, number (percent)		
e13a2	31 (25)	9 (8)
e13a2 + e14a2	6 (5)	33 (28)
e14a2	68 (56)	22 (19)
Major breakpoint, exact type unknown	17 (14)	54 (46)
Second generation TKI, given first- or second-line	28 (23%)	23 (19%)

Only the distribution of transcript types e13a2 vs. e13a2 (+e14a2), differed significantly between the two samples (P = 0.0253).

*TKI* Tyrosine kinase inhibitor, *DMR* Deep molecular remission (= BCR::ABL1 transcripts ≤0.01% on the International Scale).

^a^Missing data for 56 patients in the training and 9 patients in the validation sample.

^b^Missing data for 48 patients in the training and 6 patients in the validation sample.

^c^Missing data for 24 patients in the training and 2 patients in the validation sample.

### Master regulators driving TFR/relapse phenotypes

**Fig. 2:**
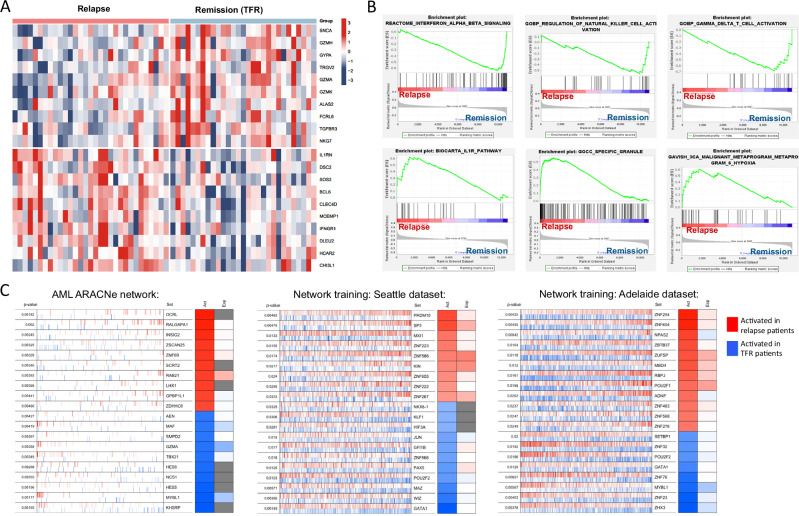
Gene expression and regulon pattern in CML patients at the last day of TKI intake. **A** Heatmap with top differential expressed genes in relapse versus TFR patients (*n* = 60). **B** Relevant gene signatures (GSEA, Broad Institute, V4.3.3) enriched in relapse or TFR patients (*n* = 60). **C** Top 20 activated regulons within the Mannheim sample (*n* = 60). Left figure: Regulons were calculated based on the AML *ARACNe* transcription factor network. Middle and right figure: Transcription factor networks were trained by the Seattle dataset (including chronic phase, accelerated phase and blast crisis patients) or the Adelaide dataset (untreated chronic phase CML at diagnosis) respectively. TFR Treatment-free remission, *ARACNe* Algorithm for the reconstruction of accurate cellular Networks.

Differential gene expression in peripheral blood leukocytes on the final day of TKI intake revealed two distinct molecular phenotypes. Peripheral blood leukocytes from TFR patients exhibited upregulation of genes typically associated with immune activation, including *GZMA, GZMH, GZMK, NKG7*, and *FCRL6*, which are primarily expressed by NK-cells and CD8 + T-lymphocytes. In contrast, relapse patients showed overexpression of both pro-inflammatory and anti-inflammatory genes in peripheral blood leucocytes (Fig. 2A). To further investigate the mechanisms within the TFR/relapse context, gene set enrichment analyses were conducted. TFR patients showed enrichment of immune activation and IFN gene signatures, whereas relapse patients were enriched in pro-inflammatory pathways (Fig. 2B).

**Fig. 3:**
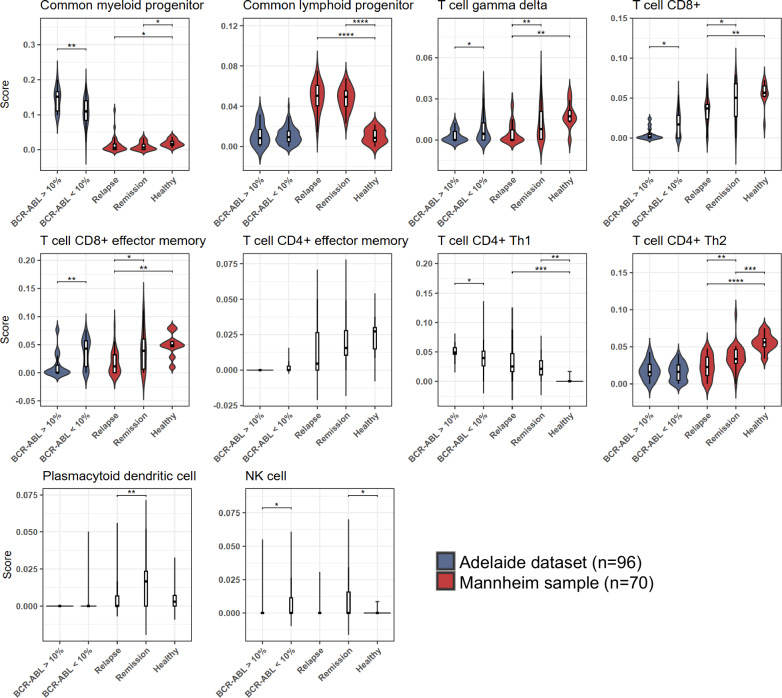
Evolution of immune cell subsets in CML patients. The relative abundance of immune cell subsets was inferred from bulk gene expression profiles using the *xCell* tool within the Mannheim sample (relapse: *n* = 31, remission: *n* = 29, healthy: *n* = 10) and the Adelaide dataset (BCR::ABL1 > 10%: *n* = 13, BCR::ABL1 ≤ 10%: *n* = 83). The Wilcoxon rank-sum test with Benjamini–Hochberg adjustment was used to assess significance within the same dataset. Significance levels are denoted as follows: *P < 0.05, **P < 0.01, ***P < 0.001 and ****P < 0.0001.

It should be noted that changes in gene expression do not necessarily reflect protein activity. Therefore, master regulators (regulons) were analyzed using the *ViPER* algorithm to better estimate protein activity [23]. Transcription factor networks were generated with the *ARACNe-AP* algorithm, utilizing in silico studies including CML patients at diagnosis for network training (see methods). The Seattle dataset included patients with chronic phase, accelerated phase and blast crisis, whereas the Adelaide dataset included only chronic phase CML at diagnosis. Regulon analysis based on the AML *ARACNe* network indicated activation of IL1RL1 in relapse patients (Fig. 3), which is consistent with the *IL1R* gene signature enrichment observed via GSEA (Fig. 2B). Also, GZMA appears again to be activated during sustained treatment-free remission. Both the “AML *ARACNe*”- and Adelaide-trained networks identified MYBL1 as activated in leucocytes of TFR patients. MYB plays a crucial role in erythropoiesis [24]. In addition, both GATA1 and KLF1 regulons were activated in patients with sustained TFR. The GATA1/KLF1 axis has been shown to be exclusively activated within the erythroid lineage [25, 26]. Multiple POU family genes showed strong activation in either relapse or TFR patients, and since the activation of these transcription factors was detected in both the Adelaide and Seattle trained networks, they may represent important regulators within the TFR/relapse ecosystem (Fig. 3).

### Immune dysregulation characterizes relapse

Immune cell scores were generated by the *xCell* enrichment algorithm. The external Adelaide dataset, including CML patients at diagnosis without prior TKI exposure, was used for comparison with the Mannheim sample (relapse: *n* = 31, remission/TFR: *n* = 29, healthy: *n* = 10). The Adelaide dataset assessed early molecular response (EMR) including 13 patients with BCR::ABL1 > 10% after 3 months of TKI treatment and 83 patients with BCR::ABL1 ≤ 10% after 3 months of TKI treatment (Fig. 3). These patients originate from the TIDEL-II trial comprising individuals who received first-line imatinib therapy [27]. We identified a distinct enrichment of myeloid progenitor cells in CML patients at the time of diagnosis (=TKI-naïve), as proof of concept. In contrast, CML patients with at least 3 years of TKI treatment showed depletion of myeloid progenitor cells and enrichment of lymphoid progenitor cells in comparison to healthy subjects (Fig. 3). In general, we observed that better responses were associated with higher lymphocyte enrichment scores. Most lymphocyte levels showed a positive correlation with response, except for Th1 CD4 + T-lymphocytes, which were negatively correlated with TKI response. Plasmacytoid dendritic cells and NK-cells displayed higher enrichments in TFR patients, but given their low enrichment scores, these findings require further independent validation. Regulatory T-cells showed low and non-significant enrichment (data not shown).

**Fig. 4:**
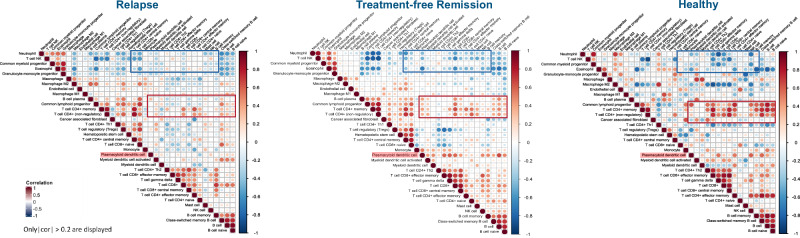
Correlation heatmaps of immune cell subsets. Correlation heatmaps of the different stromal and immune cell subsets from patients with relapse (left, *n* = 31), TFR (middle, *n* = 29) and healthy volunteers (right, *n* = 10). TFR Treatment-free remission, Cor Correlation.

Immune cell correlation plots were used as immune landscape fingerprints, to provide further insight into the TFR/relapse ecosystem (Fig. 4). In both TFR patients and healthy volunteers, positive correlations were observed between CD4 + T-lymphocytes, CD8 + T-lymphocytes, plasmacytoid dendritic cells and B-lymphocytes (Fig. 4, red box). This pattern was absent in relapse patients. Neutrophils and NKT-lymphocytes showed a negative correlation with most T-lymphocyte subsets in TFR patients and healthy volunteers (Fig. 4, blue box). However, this correlation was absent in relapse patients.

**Fig. 5:**
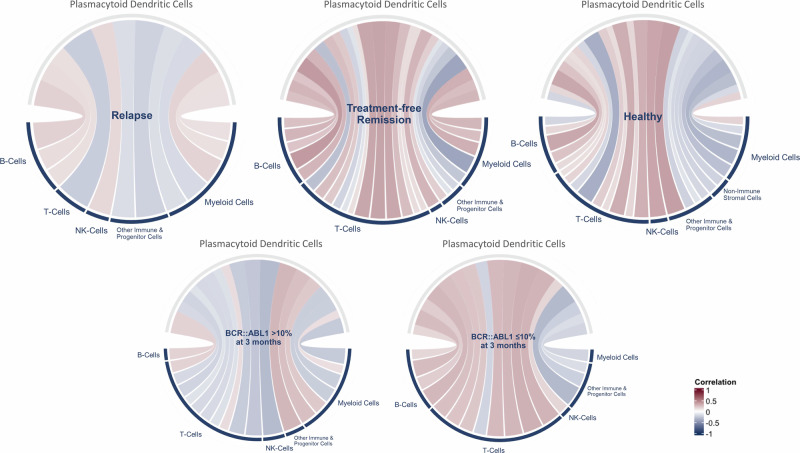
Context-dependant correlation of plasmacytoid dendritic cells with other immune cell subsets. Chord diagrams display the correlation of plasmacytoid dendritic cells with other immune cell subsets within the Mannheim sample (relapse: *n* = 31, remission: *n* = 29, healthy: *n* = 10) and the Adelaide dataset (BCR::ABL1 > 10% after 3 months of TKI: *n* = 13, BCR::ABL1 ≤ 10% after 3 months of TKI: *n* = 83). Only spearman correlations >0.2 are displayed.

Of note, plasmacytoid dendritic cells showed an exclusive and distinct positive correlation with CD4 + /CD8 + T-lymphocytes, B-lymphocytes and NK-cells in TFR patients. This trend became even more apparent in the chord diagrams (Fig. 5 & Supplementary Fig. S2). A comparable observation was made in the Adelaide dataset among CML patients at diagnosis. For patients achieving a good molecular response after three months of TKI treatment (BCR::ABL1 ≤ 10%), plasmacytoid dendritic cells correlated positively with B- and T-lymphocytes. Conversely, this correlation was lost in cases with poor molecular response following three months of TKI treatment (BCR::ABL1 > 10%).

To find a link between the immune subpopulations and TFR-specific gene pathways, we employed an unsupervised data-driven method, called *consensus independent component analysis* (*consICA*). The analysis identified a key component with higher activity in TFR patients (*P* = 0.029). This component represents a transcriptional program of adaptive and innate immunity (GO analysis, *P* < 0.001) (Supplementary Fig. S3A, B), and its activity was strongly correlated with the abundance of CD8+ effector memory T-cells (R = 0.81, *P* < 0.001) (Supplementary Fig. S3C), thus independently confirming a correlation between a specific T-cell driven transcriptional program and successful TFR.

### Prognostic influence of novel biomarkers on TFR

**Fig. 6:**
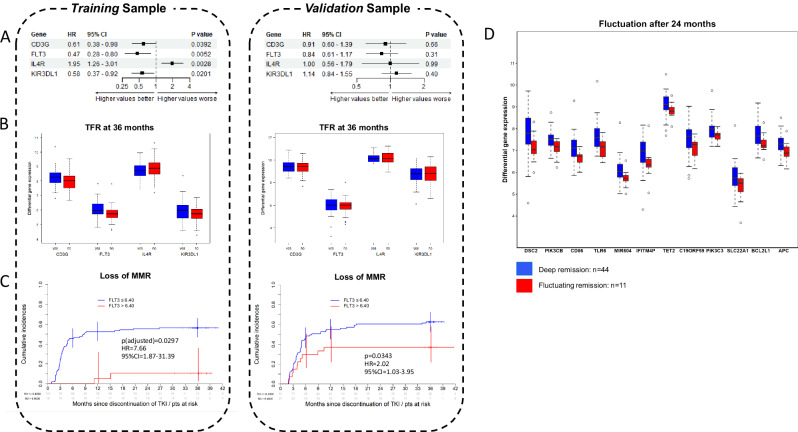
TFR/relapse biomarker analyses using training and validation sample. **A** Multiple Cox regression analysis results in 4 genes regarding their prognostic influence on treatment-free remission in both the training and the validation sample. **B** Boxplots show differential gene expressions in dependence on MMR status at 36 months in the training and the validation sample. While significant differences were observed in the training sample, Mann–Whitney U test: CD3G (*P* = 0.0073), FLT3 (*P* = 0.0020), and KIR3DL1 (*P* = 0.0434), no significant difference was found in the validation sample. **C** Cumulative incidences of MMR loss after TKI discontinuation were analyzed in the training (*n* = 122) and validation (*n* = 118) samples for FLT3, divided into two groups. Cumulative incidence curves were estimated using the cumulative incidence function, considering death and TKI restart prior to MMR loss as competing events. Vertical bars at 6, 12, and 36 months indicate the upper and lower limits of the 95% confidence intervals for the estimated incidences. **D** Boxplots illustrate differential gene expression in relation to molecular fluctuation after 24 months in the training sample (*n* = 55). Fluctuating remission: ≥50% of BCR::ABL1 positive blood samples after 24 months of TFR (*n* = 11). Deep remission: <50% of BCR::ABL1 positive blood samples after 24 months of TFR (*n* = 44). Using the Mann–Whitney U test, significant differences in gene expression were found in 12 genes: DSC2 (*P* = 0.0120), PIK3CB (*P* = 0.0120), CD86 (*P* = 0.0134), TLR6 (*P* = 0.0145), MIR604 (*P* = 0.0239), IFITM4P (*P* = 0.0292), TET2 (*P* = 0.0330), C19ORF59 (*P* = 0.0355), PIK3C3 (*P* = 0.0355), SLC22A1 (*P* = 0.0400), BCL2L1 (*P* = 0.0430), and APC (*P* = 0.0472). TFR treatment-free remission, MMR Major molecular response, TKI Tyrosine kinase inhibitor, Pts Patients, HR Hazard ratio, CI Confidence interval.

We designed a curated panel of 189 genes for absolute quantification by the NanoString nCounter including differentially expressed genes based on our array analyses and relevant genes based on current literature. This gene panel was quantified by the NanoString nCounter platform on the whole training sample (*n* = 122) at the last day of TKI intake. After background correction and quality control, 26 of 189 genes were excluded from further analyses after nSolver-based quality control and background substraction (Supplementary Table S1). In univariate Cox regression analysis, we identified 16 genes with a significant influence on the hazard of MMR loss (Supplementary Fig. S4). Of those genes, four genes were also significant in a multiple Cox model (Fig. 6A). Consistent with these time-to-event analyses in the training sample, the distribution of gene expression levels differed significantly (*P* < 0.05) between the two response groups for CD3G, FLT3 and KIR3DL1 (Fig. 6B). No significant difference was observed for IL4R (*P* = 0.063). The limited number of patients and events in the training sample precluded the development of a new prognostic model. However, using the minimal P-value approach with adjustment for multiple testing, based on the gene expression of the four genes that were significant in the multiple model, a significant cut-off of 6.40 was identified for *FLT3* (adjusted *P* = 0.0297, Fig. 6C). No significant cut-off was identified for the remaining three genes. Within bootstrap analysis, a significant threshold after *p*-value adjustment was found in 914 of the 1000 samples. Among these, the threshold 6.4 was selected in 145 cases. Only the cut-off of 5.6 was selected more frequently in the bootstrap samples (*n* = 191). The adjusted *p*-value for the cut-off of 5.6 in the training sample was 0.0463. As 6.4 was the optimal cut-off (*P* = 0.0297) in training sample and the second most frequently selected cut-off in the bootstrap samples, we chose 6.4 as the final cut-off.

Subsequently, gene expression quantification of the 16 candidate genes was performed in the 118 patients of the validation sample (Supplementary Table S2). None of the 16 genes showed a significant association with the hazard of MMR loss in the validation sample (Fig. 6A & Supplementary Fig. S4). However, consistent with the finding from the training sample, the *FLT3* cut-off of 6.40 also stratified patients in the validation sample into two groups with significantly different hazards of MMR loss (*P* = 0.0343, Fig. 6C).

### Deep versus fluctuating molecular remission

Molecular fluctuations after 24 months were examined in 55 patients from the training sample who had achieved at least MMR at 36 months and had ≥3 molecular assessments after month 24. We defined “fluctuating remission” as ≥50% of BCR::ABL1 positive blood samples after 24 months of TFR and “deep remission” as <50% of BCR::ABL1 positive blood samples after 24 months of TFR. Of these 55 patients, 44 were in deep remission while 11 showed fluctuating remission. Among the 163 genes analyzed, the expression levels of 12 genes were significantly associated with fluctuation status (Fig. 6D). *DSC2* (*P* = 0.0120), *PIK3CB* (*P* = 0.0120), *CD86* (*P* = 0.0134) and *TLR6* (*P* = 0.0145) were among the most differentially expressed genes between both groups. Of note, there was no significant difference between the two groups with respect to the number of reference genes (*ABL1* or *GUS*) detected for the BCR::ABL1 IS calculation. We did not have this kind of data in the validation sample. This is why the data has to be considered as exploratory and needs further validation by other cohorts in order to exclude accidental findings.

## Discussion

The EURO-SKI Biomarker study offers new insights into the molecular immune landscape of TFR and relapse ecosystem. Our data support the hypothesis that immune surveillance plays a critical role in maintaining TFR after TKI cessation. TFR was characterized by genes involved in cytotoxicity of NK-cells and CD8 + T-lymphocytes. These findings coincide with prior reports highlighting NK-cell involvement in TFR [28]. In addition, the pDC marker CD86 was overexpressed in patients with deep remission after TKI discontinuation (Fig. 6D). Relapse patients exhibited upregulation of both pro- and anti-inflammatory immune modulators, indicating disruption of immune surveillance. This same trend was observed by profiling immune cell enrichments via *xCell* (Fig. 3). Correlation maps revealed a preserved immune interplay based on positive correlations between T- and B-lymphocytes in TFR patients, while this interplay was lost in relapse patients (Fig. 4). This phenomenon became even more evident regarding plasmacytoid dendritic cells (pDCs) and NK-cells, which are key players in bridging innate and adaptive immunity (Fig. 5). pDCs displayed a strong and distinct positive correlation with most other immune cell subsets in TFR patients and healthy volunteers, but not in patients with loss of DMR after TKI cessation. It is of note that a similar phenomenon could be observed in CML patients at diagnosis upon stratification in early molecular responders (Fig. 5). The potential of early immune profiling in CML patients as a prognostic indicator for TFR remains to be determined. We further observed a higher abundance of lymphoid progenitor cells in patients treated with TKIs (Fig. 3). However, whether this is due to the TKI effect or constitutes a systemic immune response against CML cells remains unclear.

In a scRNA-seq study, Krishnan V. et al. reported that the transition from a myeloid to an erythroid phenotype in CML patients is associated with an optimal molecular response, given the high TKI sensitivity within the erythroid lineage [25]. In the present study, we observed notable activation of the GATA1 regulon along with KLF1 and MYBL1 in TFR patients (Fig. 2C). These regulons are known master regulators within the erythroid lineage [24, 25]. A recent study demonstrated that patients treated with hydroxyurea in early phase CML exhibit higher proportions of erythroid progenitor cells [29]. Further research is needed to determine how this myeloid/erythroid switch mechanism could be exploited to potentially increase the TFR rate.

Our transcriptomic analyses indicate that the Il1R-associated genes play a significant role in patients experiencing relapse (Fig. 2A, B). In this context, the IL1 receptor accessory protein (IL1RAP) has been identified as a rare cell-surface marker present on CML stem cells but absent in normal hematopoietic stem cells. This distinct vulnerability has already been targeted both in vitro and in vivo using anti-IL1RAP-directed CAR-T cell approaches in CML [30, 31]. Our findings further support the hypothesis that TFR patients retain effective immunogenicity against CML cells. Therefore, CAR-T cell therapy during early disease phase, may represent a promising future therapeutic strategy.

To our knowledge, none of the prognostic genetic biomarkers, described to date in literature, were validated in an independent external patient sample. In the EURO-SKI Biomarker Study, gene expression differences between TFR and relapse patients were modest, showing a maximum two-fold change in array data. As the TFR/relapse process involves a complex network of mechanisms, a gene signature-based prognostic model may be best suited for TFR prognosis or prediction. However, such a gene signature risks including a large number of genes, making its translation into the clinic extremely challenging. To date, the routine application of gene signatures is only limited to DLBCL and breast cancer [32, 33]. The aim of this study was to evaluate *individual* biomarkers for their ability to predict TFR. Through absolute quantification via the nCounter platform, *FLT3* emerged as the only gene with consistently reproducible results in both the training and validation samples (Fig. 6C). FLT3 (FMS-like tyrosine kinase 3) is an important marker of hematopoietic progenitor cells, responsible for their normal development and represents one of the most mutated genes in AML [34]. Yet, FLT3 is also known to be a critical regulator in the development and differentiation of plasmacytoid dendritic cells (pDCs) [35–37]. The FLTRL-FLT3 axis is already being leveraged in innovative anti-cancer vaccine trials, aiming to recruit and expand antigen-specific pDCs [38–41]. It seems unlikely that the FLT3 expression within this study reflects the activity of leukemic progenitor cells as patients are in DMR. Based on our immune deconvolution analyses, we hypothesize that the FLT3 axis might play a role in the maintenance of a durable pDC-mediated anti-leukemic immune response. Interestingly, a small percentage of pDCs in CML are known to express low levels of BCR::ABL and to be TKI-insensitive. However, these BCR-ABL positive pDCs keep their functionality and are able to mediate CD8 + T-cell directed anti-leukemic immunity [42]. Since functional assays were outside the scope of this biomarker study, additional in vivo and in vitro validation is required to assess the role of FLT3 in TFR maintenance. Single-cell RNA sequencing of bone marrow samples from CML patients experiencing relapse versus TFR after TKI discontinuation, would further clarify these mechanisms.

POU2F1, POU2F2 and POU2AF1 are activated transcription factors within the TFR/relapse ecosystem (Fig. 2C). These factors are implicated in key immunological processes, including plasma cell and B-cell differentiation, as well as the generation of CD4+ memory T cells [43–46]. Among them, POU2AF1 is essential for maintaining cellular viability in lymphoid cell lines [47]. Despite their known roles in lymphocyte biology, the involvement of POU2F transcription factors in the pathogenesis of CML remains unexplored.

Study limitations include the use of bulk transcriptomics, the absence of functional assays and the underrepresentation of non-European populations. The limited number of patients and events in the training sample precluded the development of a new prognostic model differentiating between risk groups. Instead, the primary aim of the study was to identify genes with a univariate significant association with TFR and to establish a multiple model with independent prognostic genes in the training sample. Subsequently, all significant genes were to be evaluated as part of a reduced gene panel in the validation sample, with adjustment for multiplicity based on the number of genes tested. We acknowledge that further validation in a bigger cohort is needed for the evaluation of FLT3 within a new prognostic model.

In conclusion, the findings of this study indicate that TFR patients maintain persistent communication between innate (NK cells, pDCs) and adaptive immunity (e.g., CD8+, CD4+, and γδ T lymphocytes), mirroring patterns observed in healthy controls. This intercellular communication is disrupted in relapse patients. Single-cell RNA sequencing analysis of bone marrow is critical for elucidating the pathogenetic mechanisms underlying this immune dysregulation. Our findings suggest that immune system priming may occur in the early CML disease stage. Furthermore, we identified an FLT3 threshold that distinguished two patient groups with significantly different probabilities of TFR loss. The role of FLT3 in leukemia seems to be a double-edged sword. Thus, functional assays are necessary to clarify the role of FLT3 in sustaining a CML-specific immune response. Leveraging mechanisms that reestablish or reinforce communication between innate and adaptive immunity could be pivotal in achieving durable TFR.

## Supplementary information

Supplemental Material

Table S1

Table S2

## Data Availability

ArrayExpress ID: E-MTAB-16324.
