# Supplementary material for: Comprehensive molecular characterization of treatment-free remission and molecular relapse in chronic myeloid leukemia patients: the EURO-SKI Biomarker Study
Source: Leukemia. 2026 Jun 18;40(8):1698–707. doi: 10.1038/s41375-026-03001-5 (PMC13421316; doi:10.1038/s41375-026-03001-5)
Supplement: Supplementary file 1 — Supplemental Material [file 41375_2026_3001_MOESM1_ESM.pdf]

Supplementary Tables Legends

Table S1

Training Sample nCounter Normalized Gene Expression Matrix

Table S2

Validation Sample nCounter Normalized Gene Expression Matrix

Supplementary Figures

Figure S1

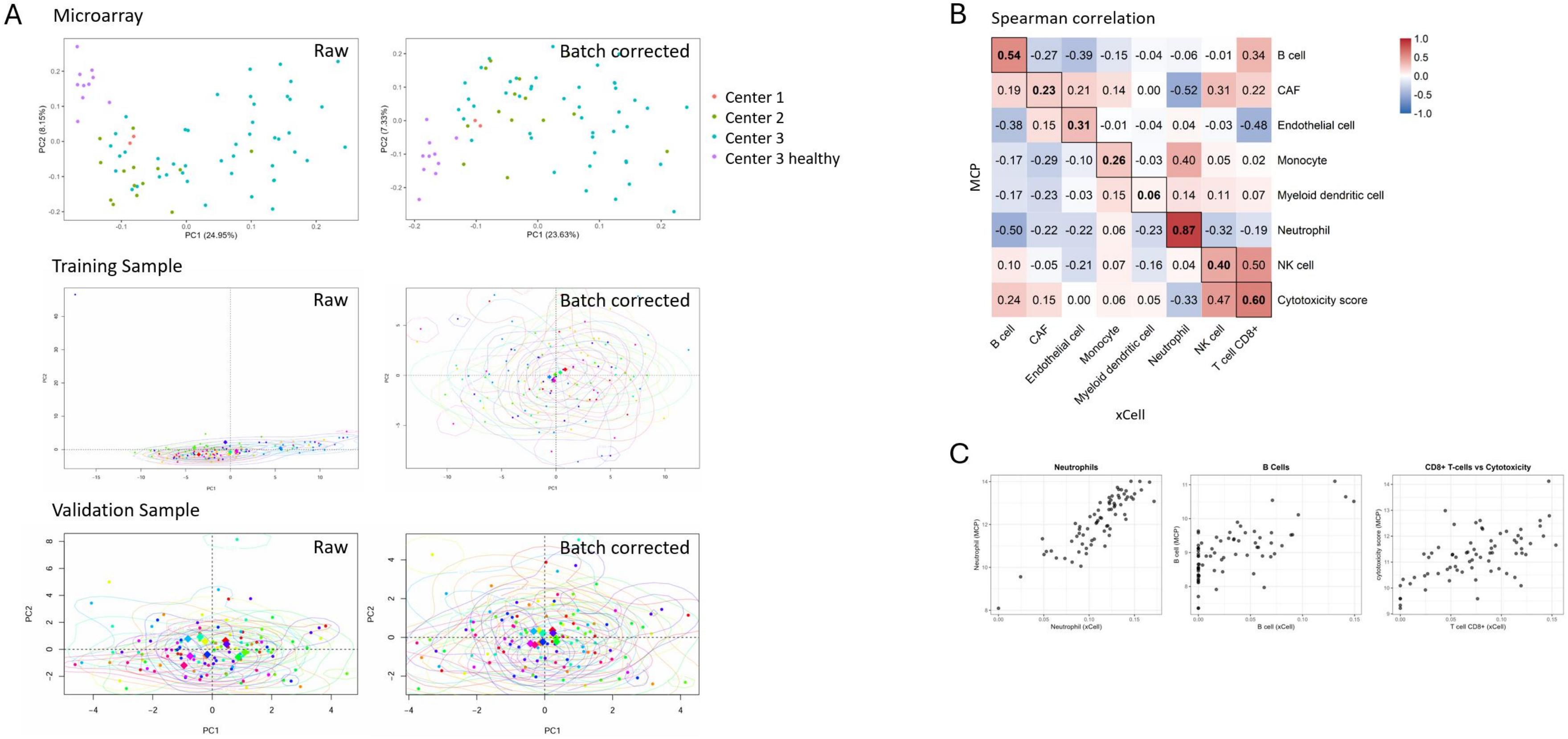

A. Principal Component Analysis of transcriptomic profiles before (Raw) and after (Batch corrected) batch effect adjustment across the Microarray, Training, and Validation datasets. ComBat was applied to correct the technical clustering of outlier samples originating from

Medical Center 2. Similarly, batch correction within the nCounter datasets was performed to adjust for outlier cartridges. This successfully mitigates technical center- and cartridge-specific variance.

B. Heatmap showing Spearman correlation of immune cell deconvolution scores between xCell and MCP-counter.

C. Concordance between xCell and MCP-counter deconvolution scores for selected immune cells.

CAF: cancer-associated fibroblasts

Figure S2

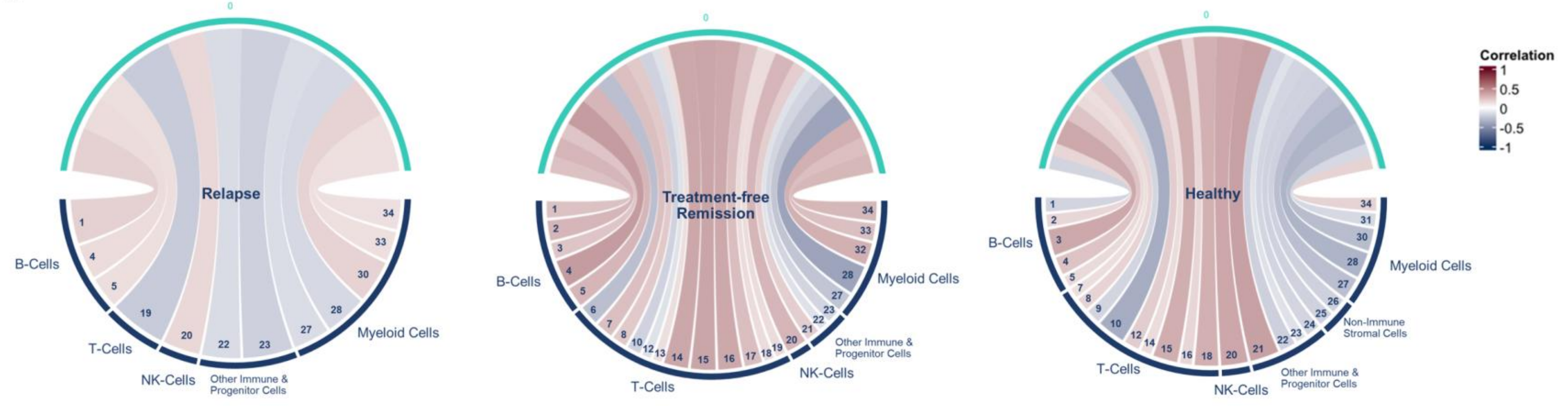

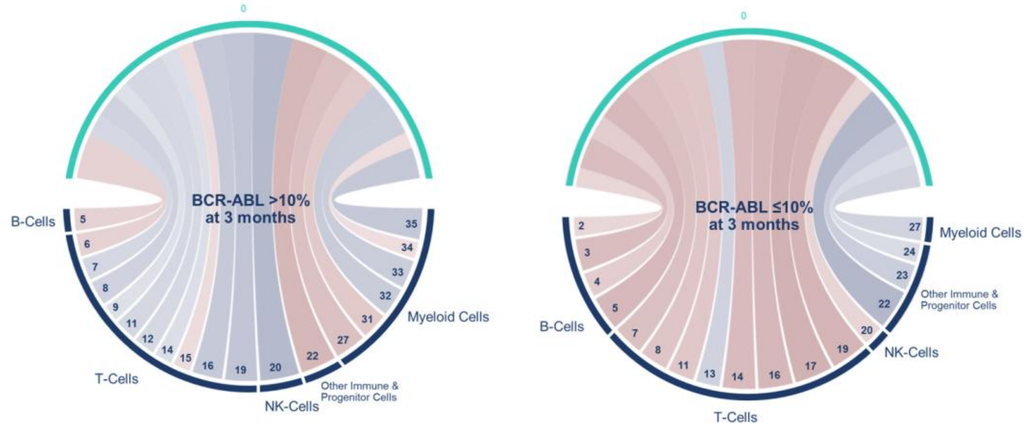

Num.	Cell type	Num.	Cell type
0	Plasmacytoid dendritic cell	18	T cell CD4+ effector memory
1	B cell plasma	19	T cell CD4+ naive
2	B cell memory	20	NK cell
3	Class-switched memory B cell	21	Common lymphoid progenitor
4	B cell	22	Common myeloid progenitor
5	B cell naive	23	Granulocyte-monocyte progenitor
6	T cell NK	24	Hematopoietic stem cell
7	T cell CD4+ memory	25	Endothelial cell
8	T cell CD4+ (non-regulatory)	26	Cancer associated fibroblast
9	T cell CD4+ Th1	27	Neutrophil
10	T cell regulatory (Tregs)	28	Eosinophil
11	T cell CD4+ central memory	29	Macrophage
12	T cell CD8+ naive	30	Macrophage M2
13	T cell CD4+ Th2	31	Macrophage M1
14	T cell CD8+ effector memory	32	Monocyte
15	T cell gamma delta	33	Myeloid dendritic cell activated
16	T cell CD8+	34	Myeloid dendritic cell
17	T cell CD8+ central memory	35	Mast cell

Chord diagrams display the interplay of plasmacytoid dendritic cells with other immune-cell subsets within the Mannheim sample (relapse: n=31, remission: n=29, healthy: n=10) and the Adelaide dataset (BCR::ABL1 >10% after 3 months of TKI: n=13, BCR::ABL1

≤10% after 3 months of TKI: n=83). Only spearman correlations >0.2 are displayed. The legend for the numbered cell types is provided on the table.

Figure S3

A

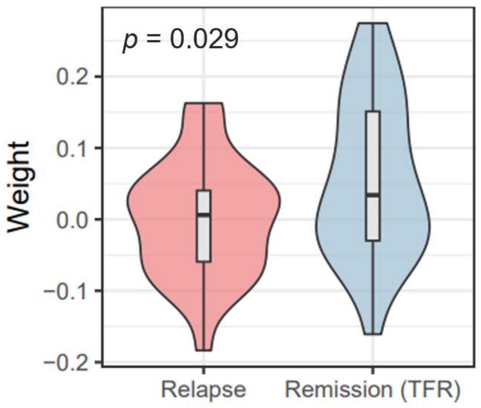

B

GO ID	Term	FDR
GO:0002250	adaptive immune response	7.92e-06
GO:0002717	positive regulation of NK cell mediated immunity	1.78e-05
GO:0006968	cellular defense response	4.84e-05
GO:0006955	immune response	3.30e-04
GO:0007186	G protein-coupled receptor signaling pathway	1.69e-03
GO:0031640	killing of cells of another organism	8.14e-03
GO:0019722	calcium-mediated signaling	8.49e-03
GO:0032814	regulation of natural killer cell activation	1.16e-02
GO:0150076	neuroinflammatory response	1.39e-02
GO:0050852	T cell receptor signaling pathway	1.45e-02

C

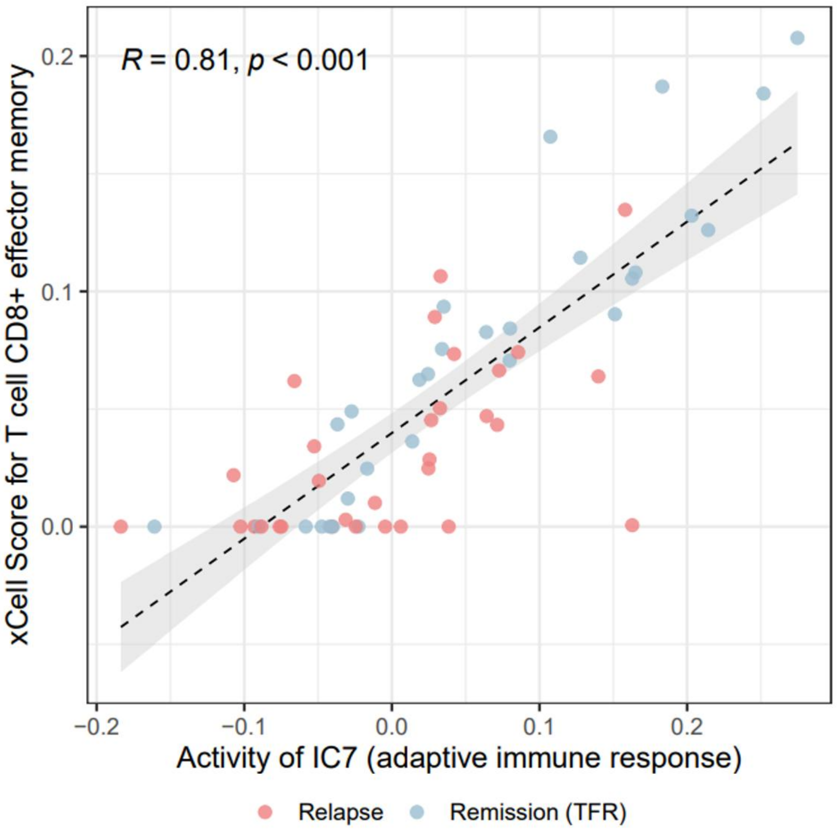

A. Violin plot showing the distribution of Independent Component 7 activity scores in relapse (n=31) and treatment-free remission (n=29) patient groups.

B. Table of the top 10 enriched Gene Ontology (GO) biological processes based on genes significantly contributing to ICA Component 7.

C. Scatter plot showing the positive correlation between the activity of Component 7 and the xCell-derived enrichment score for CD8+ effector memory T-cells. Spearman's rank correlation coefficient (R) and p-value are displayed.

TFR: Treatment-free remission, FDR: False discovery rate, ICA: Independent component analysis, IC7: Independent component 7

Figure S4

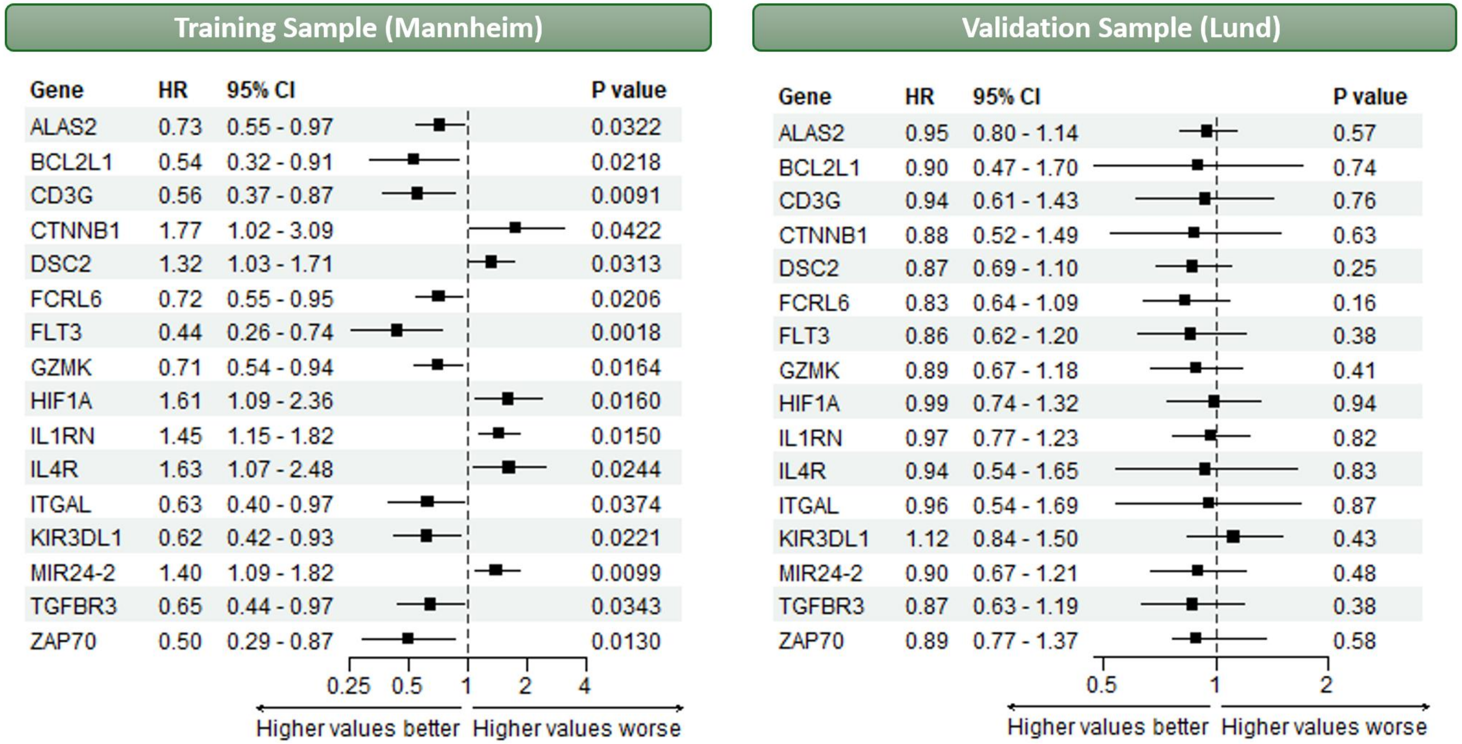

Univariate Cox regression analysis results of 16 genes regarding their prognostic influence on treatment-free remission in both the training and the validation sample.

HR: Hazard ratio. CI: Confidence interval.
